# Notes on Pauropoda (Myriapoda) from USA, with descriptions of two new species

**DOI:** 10.3897/zookeys.115.1190

**Published:** 2011-07-05

**Authors:** Ulf Scheller

**Affiliations:** Häggeboholm, Häggesled, 53194 Järpås, Sweden

**Keywords:** Myriapoda, Pauropoda, new species, taxonomy, USA

## Abstract

Two new species of Pauropoda are described from USA, *Kionopauropus alyeskaensis* **sp. n.** (Pauropodidae), and *Eurypauropus arcuatus* **sp. n.** (Eurypauropodidae). The genus *Kionopauropus* is reported from the Western Hemisphere for the first time.

## Introduction

The collection of Pauropoda lodged in the Department of Entomology, Smithsonian Institution, Washington, has been studied. The specimens are preserved in alcohol, most specimens are old, opaque and/or covered by small particles and not possible to recognize. The species which could be identified are treated below.

Abbreviations: ad., subad., and juv. = an adult, a subadult or juvenile specimen with the number of pairs of legs indicated.

Measurements: length of body in mm, otherwise the text refers to relative lengths (reference value in text); range of variation in adult paratypes given in brackets. Quotations from labels are given in inverted commas.

## Systematics

### **Family Pauropodidae Genus** Pauropus **Lubbock, 1867**

In 1896 O.F. Cook described *Pauropus bollmani* from specimens in the National Museum collected by C.H. Bollman. The description is meager and this species has long been considered as species *incertae sedis*. Cook’s material seems to be five specimens now in a vial with four labels “*Pauropus bollmani* Cook Bloomington, Indiana Bollman, no. 112”, “Type”, “Type 3213” and “USNM 2053511”. The specimens are brown and opaque and more or less defect (antennae, many legs, most bothriotricha lost, body two-parted or crushed). None of the specimens (three adults, two females and one male, and two ones stad.?) could be identified. *Pauropus bollmani* Cook has still to be on the list of species *incertae sedis*.

#### Pauropus huxleyi

Lubbock, 1867

##### Material.

 USA, Pennsylvania, Spring Brook Twp., Expt. no. C4b, 1 ad. (♀), 1945.v.12, and same locality, no. C4e, 6 ad. (1♂, 5♀), 1 juv. 5, both R.I. Sailor leg.

### Genus Kionopauropus Scheller, 2009

The genus was erected for three species from Indonesia and the Philippines (Scheller 2009). At least two more species have to be included here, *Kionopauropus* (ex *Decapauropus*) *facetus* (Remy, 1956) comb. n. from Madagascar and *Kionopauropus* (ex *Decapauropus*) *lituiger* (Remy, 1957) comb. n. from West Australia, both with the anterodistal corner of the sternal antennal branch more truncated than the posterodistal corner, the antennal globulus *g* with long stalk and the pygidial sternum with the setae combination *b*1+*b*2. With the species described below the genus has six species distributed from Madagascar and Australia in the South to Alaska in the North. It probably occurs also in Japan (Y. Hagino, Natural History Museum and Institute, Chiba Museum, Japan, pers. com.).

#### Kionopauropus alyeskaensis sp. n.

urn:lsid:zoobank.org:act:C6931196-AB1D-4C90-B42C-69017D41A2CD

http://species-id.net/wiki/Kionopauropus_alyeskaensis

Figs 1–7 

##### Type material.

 Holotype, ad. 9 (♀), USA, Alaska, 15 mls N of Fairbanks, 1949.iv.28, S. Lienk leg.

##### Etymology.

 Latinised from the old Aleut word ‘alyeska’, meaning mainland (referring to the collecting site on the Alaskan mainland).

##### Diagnosis.

 *Kionopauropus alyeskaensis* n. sp. seems to be in a group of its own because it has long and slender antennal branches and 4-parted anal plate, characters not found in species described up to now.

##### Description.

**Figures 1–7.**
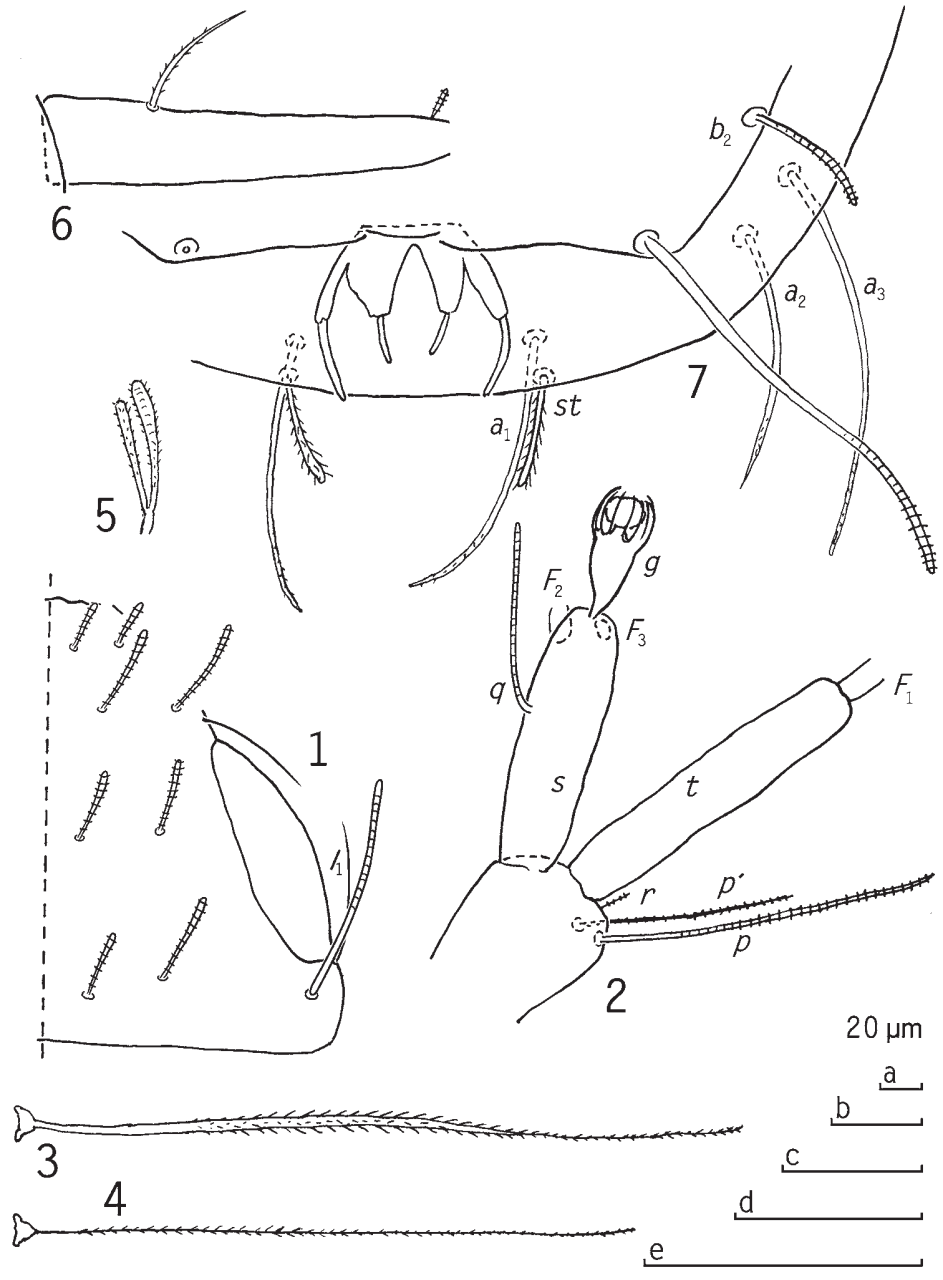
*Kionopauropus alyeskaensis* sp. n., holotype ad. 9 (♀) **1** head, median and right part, tergal view **2** right antenna, inner view **3** *T*3 **4** *T*5 **5** seta on trochanter of leg 9 **6** tarsus of leg 9 **7** pygidium, sternal view. Scale bars: a: 4; b: 1, 3, 6; c: 2; d: 5; e: 7.

 Length. ?, specimen two-parted. Head (Fig. 1). Setae of medium length, striate, tergal ones somewhat clavate, lateral ones cylindrical. Relative lengths of setae, 1st row: *a*1=*a*2=10; 2nd row: *a*1=16, *a*2=17, *a*3=20; 3rd row: *a*1=12, *a*2=13, 4th row: *a*1=12, *a*2=15, *a*3=?, *a*4=37; lateral group setae: *l*1=25, *l*2=20, *l*3=?. Ratio *a*1/*a*1-*a*1 not possible to measure. Temporal organ in tergal view narrow, short, ≈0.8 of shortest interdistance; pistil and pore not ascertained. Head cuticle glabrous.

Antennae (Fig. 2) not complete, on segment 4 only three setae found, all thin, cylindrical, striate, their relative lengths: *p*=100, *p’*=67, *r*=10. *p* as long as tergal branch *t*, the latter cylindrical, 5.2 times as long as greatest diameter and 1.2 times as long as sternal branch *s*. The latter branch 3.6 times as long as greatest diameter, anterodistal corner more truncate than posterodistal one. Seta *q* thin, cylindrical, striate, 0.7 of the length of *s*. Flagella lost. Globulus *g* longish, with stalk included 2.7 times as long as wide, 0.5 of the length of *s*, diameter of *g* 0.9 of greatest diameter of *t*, ≈11 bracts, capsule subspherical. Antennae glabrous.

Trunk. Collum segment hidden. Setae on tergites only partly available for study, those studied thin, cylindrical, proportionately long.

Bothriotricha (Figs 3, 4). *T*1, *T*2 and *T*4 lost; proximal 2/3 of *T*3 (Fig. 3) with thickened axes with distinct oblique pubescence, distal 1/3 very thin with short pubescence; *T*5 (Fig. 4) thin with short pubescence.

Legs (Figs 5, 6). Setae on coxa and trochanter (Fig. 5) of leg 9 furcate, main branch clavate, secondary branch subcylindrical, a little shorter than main branch, both branches with short oblique pubescence. Tarsus of leg 9 (Fig. 6) straight, tapering, 4.7 times as long as greatest diameter; proximal seta outstanding, curved, tapering, pointed, with depressed pubescence, 4 times longer than straight, cylindrical, blunt, striate distal seta. Cuticle of tarsus glabrous.

Pygidium (Fig. 7).

Tergum. Posterior margin rounded. a-group setae thin, tapering, pointed, curved inward; *st* cylindrical, blunt, distinctly pubescent, curved inward and converging; relative lengths of setae: *a*1=10, *a*2=9, *a*3=13, *st*=4. Distance *a*1-*a*1 0.8 of the length of *a*1, distance *a*1-*a*2 3.3 times as long as distance *a2*-*a3*, distance *st*-*st* 2.3 times as long as *st* and inconsiderably longer than distance *a*1-*a*1.

Sternum. Posterior margin with shallow indentation below anal plate. Relative lengths of setae (pygidial *a*1=10): *b*1=15, *b*2=5. Both setae cylindrical, blunt, striate.

Anal plate 4-branched by three deep posterior V-shaped incisions, median one deepest and broadest, lateral branches cylindrical, submedian branches triangular, cut squarely distally; each branch with cylindrical appendages projecting backward, those of inner branches about 0.5 of the length of those on lateral branches, appendages curved inward; plate and appendages glabrous.

### Family Eurypauropodidae. Genus Eurypauropus Ryder, 1879

#### Eurypauropus spinosus

Ryder, 1879

##### Material.

 USA, Indiana, Bloomington, 37 ad. (36♀, 1 sex?), 1 ?ad.(sex?), 2 subad. 8(♀), 2 stad. ?(sex?), “*Eurypauropus* 93 Bollm. Col.?”.

#### Eurypauropus arcuatus sp. n.

urn:lsid:zoobank.org:act:D0DED3CE-2557-4CEE-B55E-BF29EF85EE14

http://species-id.net/wiki/Eurypauropus_arcuatus

Figs 8–17 

##### Material.

 Holotype, ad. 9(♀), USA, Virginia, Fairfox County, Dead Run, 1948.x.10, E.A. Chapin leg. – Paratype, 1 ad. (♀), same data as holotype. – Non type material, 1 subad. 8 (♀), same data as holotype.

##### Etymology.

 From the Latin ‘arcuatus’ = bend like a bow (referring to the curved antennal globulus *g*).

##### Diagnosis.

 *Eurypauropus arcuatus* is close to the two species earlier known from North America, *Eurypauropus spinosus* Ryder, 1879, and *Eurypauropus washingtonensis* Scheller, 1985, the former wide-spread in USA, the latter known from National Olympic Park in Washington only. The new species is distinguished from both these species by the shape of the antennal globuli, *g*, curved in *Eurypauropus arcuatus*, not straight, globulus *g’* of 3rd antennal segment, as long as wide, not at least twice longer than wide, by the shape of the sternal antennal branch *s*, anterior and posterior margins subsimilar in length, not the posterodistal margin distinctly shorter than the anterodistal one, by the shape of the setae *st* of the pygidial tergum, simply curved inwards, not *S*-shaped, and by the shape of the anal plate, no posterolateral appendages, distinct in both *Eurypauropus spinosus* and *Eurypauropus washingtonensis*.

##### Description.

**Figures 8–17.**
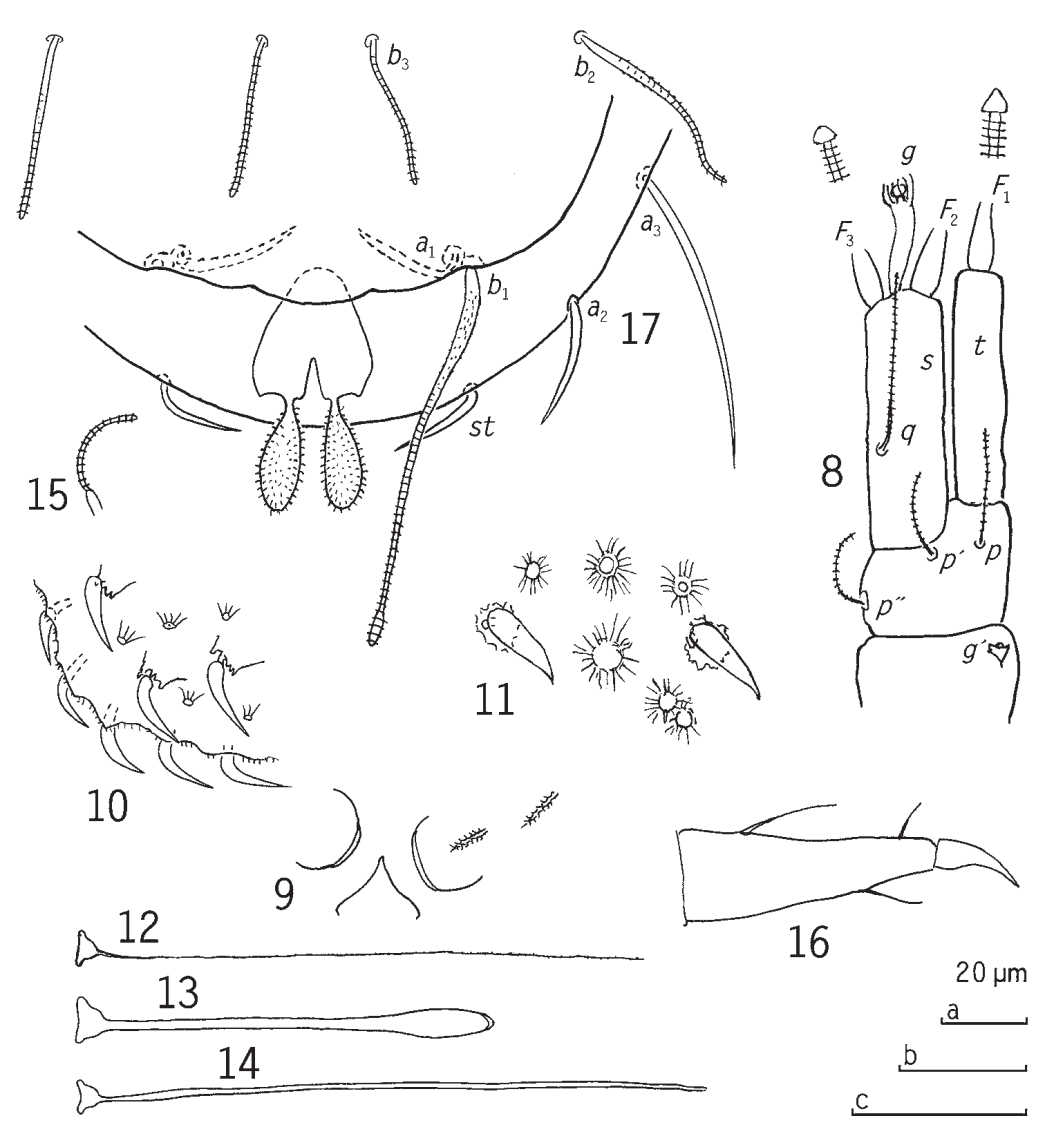
*Eurypauropus arcuatus* sp. n., holotype ad. 9 (♀) **8** right antenna, sternal view **9** collum segment, median and left part, sternal view **10** tergite II, left posterior corner with large setose and small crater-like protuberances, lateral view **11** tergite I, central part, large setose and small crater-like protuberances, tergal view **12** *T*1 **13** *T*3 **14** *T*5 **15** seta on trochanter of leg 9 **16** tarsus of leg 9 **17** pygidium, sternal view. Scale bars: a: 8-10, 12-15; b: 11; c: 16, 17.

 Length 1.37–1.42 mm. Antennae (Fig. 8) glabrous. Segment 3 with two setae and globulus *g’*, segment 4 with three setae. Globulus *g’* short, as long as wide. Relative lengths of setae on 4th segment: *p*=100, *p’*=84, *p”*=88. These setae cylindrical, pointed, striate. Tergal seta *p* 0.5 of the length of tergal branch *t*. The latter cylindrical, 4.5 times as long as greatest diameter and as long as sternal branch *s*. The latter branch cylindrical, 4.0 times longer than greatest diameter, anterodistal and posterodistal corners equally truncate; its seta *q* as setae similar to those of segment 4, and inserted 0.4 from proximal end of *s*, with length 0.7 of the length of *s*. Relative lengths of flagella (base segments included) and base segments: *F*1=100, *bs*1=(10-)11, *F*2=66, *bs*2=10, *F*3=85(-87), *bs*3=12. *F*1 0.9 of the length of *t*, *F*2 and *F*3 1.3 and 1.7 times as long as *s* respectively. Distal calyces of flagella conical, glabrous. Globulus *g* slender, 4 times longer than wide, stalk thin, curved, ≈10 thin bracts; diameter of *g* 0.6 of greatest diameter of *t*.

Trunk. Setae of collum segment (Fig. 9) short, probably simple, subsimilar, short, tapering, pointed, densely pubescent. Sternite process triangular, pointed anteriorly. Appendages short, wide, caps thin. Process and appendages glabrous.

Tergites with two types of protuberances (Figs 10, 11), large, curved, setose, glabrous, pointed spines, proximally scale-like and evenly spaced on background of more numerous, small crater-like protuberances with pleated sides.

Bothriotricha (Figs 12–14). Relative lengths: *T*1=100, *T*2=97(-112), *T*3=67(-71), *T*4=117, *T*5=102(-104). *T*1 (Fig. 12) and *T*2 with very thin axes, distal half with short erect pubescence; *T*3 (Fig. 13) glabrous, with thick axes and distal longish end-swelling, *T*4 and *T*5 (Fig. 14) glabrous too but thin, somewhat tapering, blunt distally.

Legs (Figs 15–16). All legs 5-segmented. Setae on coxa and trochanter (Fig. 15) of leg 9 thin, simple, cylindrical, striate. Tarsus of leg 9 distinctly tapering (Fig. 16), (2.8-)3.2 times as long as greatest diameter, two tergal setae and one sternal, all pointed glabrous; proximal tergal seta longest, almost 0.4 of the length of tarsus, (1.8-)2.2 times as long as distal tergal seta and 1.6 times as long as sternal seta. Length of main claw 0.4 of the length of tarsus.

Pygidium (Fig. 17).

Tergum. Posterior margin evenly rounded. Relative lengths of setae: *a*1=*a*2=10, *a*3=(22-)23, *st*=7. *a*1, *a*2 and *st* similar, tapering, pointed, *a1* and *st* curved inwards with a knee close to proximal end, *a*2 evenly curved, *a*1 and *st* strongly and *a*2 inconsiderably converging; *a*3 evenly curved inward and somewhat diverging. Distance *a*1-*a*1 (2.1-)2.4 times as long as *a*1, distance *a*1-*a*2 about as long as distance *a*2-*a*3; distance *st*-*st* 3.2 times as long as *st* and 1.1 times as long as distance *a*1-*a*1.

Sternum. Posterior margin with low posteriomedian bulge below anal plate. Relative lengths of setae (pygidial *a*1=10): *b*1=(23-)27, *b*2=13 and 16 in the holotype, and 18 in the paratype, *b*3=12. *b1* with fusiform, and shortly pubescent base, tapering into a subcylindrical, striate distal half terminated with a small, striate end-swelling; *b2* similar to *b*1 but with cylindrical distal part, proportionately much shorter and without end-swelling; *b*3 subcylindrical, striate. Length of *b*1 (as long as -)1.2 times as long as interdistance, *b*2 0.7-0.9 of distance *b*1-*b*2, *b*3 on the same level as *b*2, length 1.4 times as long as interdistance.

Anal plate narrowest at base and consisting of two broad lobes posteriorly separated by V-shaped incision ≈0.3 of the length of plate; posterolateral corners without appendages but each with very small tooth; each lobe with a bladder-shaped posteriorly directed appendage with distinct erect pubescence, length of appendages 0.9 of the length of plate.

## Remarks

The Pauropoda was reported from USA for the first time by Packard (1870) only four years after the group was discovered in London in 1866 (Lubbock 1867). Despite that more than 140 years have passed and about one hundred species have been found the knowledge of their occurrence in the USA is still most imperfect.

## Supplementary Material

XML Treatment for Pauropus huxleyi

XML Treatment for Kionopauropus alyeskaensis

XML Treatment for Eurypauropus spinosus

XML Treatment for Eurypauropus arcuatus
